# Oculometric variations during mind wandering

**DOI:** 10.3389/fpsyg.2014.00031

**Published:** 2014-02-11

**Authors:** Romain Grandchamp, Claire Braboszcz, Arnaud Delorme

**Affiliations:** ^1^Centre de Recherche Cerveau et Cognition, Paul Sabatier UniversityToulouse, France; ^2^Centre de Recherche Cerveau et Cognition, CNRS, UMR5549Toulouse, France; ^3^Swartz Center for Computational Neuroscience, Institute for Neural Computation, University of California San DiegoLa Jolla CA, USA

**Keywords:** mind wandering, pupil size, blinks, gaze position, classification

## Abstract

A significant body of literature supports the contention that pupil size varies depending on cognitive load, affective state, and level of drowsiness. Here we assessed whether oculometric measures such as gaze position, blink frequency and pupil size were correlated with the occurrence and time course of self-reported mind-wandering episodes. We recorded the pupil size of two subjects engaged in a monotonous breath counting task while keeping their eyes on a fixation cross. This task is conducive to producing mind-wandering episodes. Each subject performed ten 20-min sessions, for total duration of about 4 h. Subjects were instructed to report spontaneous mind-wandering episodes by pressing a button when they lost count of their breath. After each button press, subjects filled in a short questionnaire describing the characteristics of their mind-wandering episode. We observed larger pupil size during the breath-focusing period compared to the mind-wandering period (*p* < 0.01 for both subjects). Our findings contradict previous research showing a higher baseline pupil size during mind wandering episodes in visual tasks. We discuss possible explanations for this discrepancy. We also analyzed nine other oculometric measures including blink rate, blink duration and gaze position. We built a support vector machine (SVM) classifier and showed that mean pupil size was the most reliable predictor of mind wandering in both subjects. The classification accuracy of mind wandering data segments vs. breath-focusing data segments was 81% for the first subject and 77% for the second subject. Additionally, we analyzed oculometric measures in light of the phenomenological data collected in the questionnaires. We showed that how well subjects remembered their thoughts while mind wandering was positively correlated with pupil size (subject 1, *p* < 0.001; subject 2, *p* < 0.05). Feelings of well being were also positively correlated with pupil size (subject 1, *p* < 0.001; subject 2, *p* < 0.001). Our results suggest that oculometric data could be used as a neurocognitive marker of mind-wandering episodes.

## Introduction

As humans, our existence in this world is both the most natural yet complex thing we experience. Technological developments have brought new ways to study, quantify and characterize the mind through brain imaging. Among the many different aspects of cognition, attention has been at the center of a long line of research and a central process in the building of our stream of consciousness (Baars, 1993, 1997; Posner, 1994; Posner and Rothbart, 1998; Simons and Chabris, 1999; Koch and Tsuchiya, 2007). Most studies of attention have focused on an externally oriented type of attention, using exteroceptive tasks such as detection tasks, categorization tasks, and working memory tasks. Internal mental events, such as phenomenological experiences of feelings, thoughts, and body sensations that are not directly triggered by our environment, are dimensions of attention that have not yet been extensively studied. In particular, spontaneous thoughts often attract our attention, supplanting perception of the external world (Smallwood et al., 2008; Schooler et al., 2011). When engaged in a task we are familiar with (especially when it does not require high attentional engagement), we are more likely to switch to a mental state which is now commonly referred to as mind wandering (MW) (Smallwood and Schooler, 2006).

Finding the neural or physiological markers of MW has become important in order to better characterize and understand this mental state. However, studying this phenomenon is not a trivial task, as it is by its very nature a spontaneous, task-unrelated, internal mental process of which the subject is often unaware. It is thus difficult to study, document and replicate mind wandering using classical psychophysical paradigms (Gruberger and Zangen, 2011). Despite these difficulties, MW has been characterized in several ways: behavioral measures (Smallwood and Schooler, 2006; Smallwood et al., 2008), EEG (Smallwood et al., 2008; Braboszcz and Delorme, 2011), fMRI (Smith et al., 2006; Christoff et al., 2009), and pupil size (Smallwood et al., 2011). Smallwood et al. (2011) showed a reduced entrainment of pupil dilation to external stimuli during mind wandering occurring during a choice reaction time task and a working memory task. They also observed increased pupil diameter during periods where mind wandering was more likely to occur. We were interested in determining whether the same effect (in addition to other potential oculometric changes) occurred during self-reported MW episodes.

There are two main reasons for our study. First, previous oculometric research on mind wandering relied on probed mind wandering protocols where subjects are asked at regular intervals if they are mind wandering. We were interested in verifying that self caught mind wandering episodes showed the same kind of oculometric effects. Also, Smallwood et al. (2011) focused on pupil size only, while we believe there are additional eye metrics that may potentially correlate with mind wandering. Second, the two tasks involved in Smallwood and colleagues' study were a choice reaction time task and working memory task. We wanted to investigate a more interoceptive concentration task, i.e., focusing on counting breath cycles, and self-monitoring of mind wandering. The choice of this task is related to the study of mind wandering in the context of meditation, similar to work done by Braboszcz and Delorme (2011), and Hasenkamp et al. (2012).

Our self-caught mind wandering protocol as described in Braboszcz and Delorme (2011) is original for two main reasons: first, it includes spontaneous meta-conscious events, (i.e., when the participant notices he or she is mind wandering) and the neuro-cognitive processes underlying meta-conscious events are poorly understood. Second, this type of protocol requires the self-monitoring of attention (and more generally of mental content) which is involved in various situations and tasks in our daily lives, and more specifically in attention training practices such as meditation. Two recent studies have shown that self-reported mind wandering paradigms can be used to characterize EEG (Braboszcz and Delorme, 2011) and fMRI (Hasenkamp et al., 2012) correlates of mind wandering in the context of a breath counting task. Here, we study for the first time oculometric correlates in the context of this task. In our task, subjects are not probed at a regular interval about their state of mental focus. Instead, they are asked to perform a breath counting task while keeping their eyes on a fixation cross, and are instructed to press a button whenever they realize they are not performing the breath counting task (i.e., they were mind wandering) (Braboszcz and Delorme, 2011). This breath counting task is similar to meditation practices that focus on the breath. This task is more interoceptive, and may be more ecological (similar to reading tasks used in previous research) than other mind-wandering psychophysics tasks.

While oculometric variations during mind wandering have not been extensively studied, we believe it may be an important physiological marker of this mental state. More specifically, pupil size variation has been associated with loecus coerelus (LC) activity (Usher et al., 1999; Carter et al., 2010), mainly through the noerepinephrine circuit. Neural activity in the noradrenergic locus coeruleus correlates with periods of wakefulness and arousal (Carter et al., 2010). It has also been shown that electrotonic coupling in noradrenergic LC neurons may play an important role in attentional modulation, and the regulation of goal-directed vs. exploratory behaviors (Usher et al., 1999).

In order to study oculometric traits and differences between mind wandering and states of concentration, we conducted statistical analysis on pupil size variation, eye blinks and gaze position. This standard statistical analysis was complemented by a classification analysis aimed at assessing the potential of using oculometric measures to detect mind wandering at the single trial level. Finally, psychometric data pertaining to self-evaluation of mind wandering episodes are reported and analyzed in light of the oculometric data.

## Materials and methods

### Subjects

Two participants S1 female (age 25) and S2 male (age 31) volunteered for this experiment after providing written informed consent. Both participants had normal or corrected to normal vision. They were both right handed and reported no mental or neurological disorder. The two subjects did not receive any monetary compensation for their participation. The experimental protocol was approved by the local ethical committee (CPP 2010-A00744-35). Both subjects had performed the task before and had been practicing meditation.

### Stimuli and procedure

**Figure 1:**
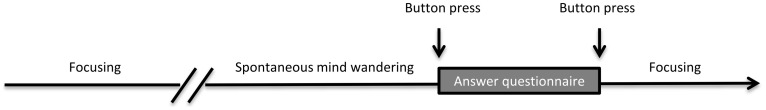
**Experimental task.** Subjects performed a breath counting/focusing task until they spontaneously started to mind wander. Upon realizing they were mind wandering (i.e., that they had lost the count of their breath), they pressed a button. They then filled a short questionnaire indicating the content of their mind wandering episode and pressed a button to resume the task.

The experimental task was adapted from Braboszcz and Delorme (2011). Subjects sat in a dimly lit room with their heads on a chin rest. A computer screen was placed 60 cm in front of them. Stimuli presentation was performed using the MATLAB platform (The MathWorks) and the Psychophysics Toolbox 3 (Brainard, 1997; Pelli, 1997). Instructions were displayed at the beginning of each session on the screen. We asked subjects to gaze continuously at a fixation cross displayed in the center of the screen. The task of the subjects was to count backward each of their breath cycles (inhale/exhale) from 10 to 1. At 1, they were instructed to restart counting backward from 10. This backward counting task was adopted as some subjects reported being able to count forward and mind wander simultaneously (Braboszcz and Delorme, 2011). We will refer to this mental state as the F mental state for Focusing. Subjects also had to indicate whenever they realized they had lost track of their breath count (i.e., that their attention had drifted) by pressing the left mouse button. Immediately following the button press, a short 1-page phenomenological questionnaire was presented on the computer screen. The questionnaire allowed the subject to characterize their mind wandering episodes—it usually took subjects less than 30 s to complete the questionnaire (average 28 ± 12 s). Subjects were required to answer each question. The graphic user interface, instructions to subjects and questionnaire content are described in supplementary materials (Supplementary Figures 1 and 2). Each question was explained to the subject during a preliminary test, allowing the subject to familiarize himself both with the questions and the user interface. After the questionnaire was completed, subjects had to press the right mouse button to indicate they were ready to restart the breath counting task. The experimental task is illustrated in Figure 1.

EEG data were recorded using a 64-channel Biosemi Active Two system. These data will not be analyzed in this report. While performing the breath counting task, subjects were also presented with a passive auditory oddball protocol that they were instructed to ignore (Braboszcz and Delorme, 2011). The subjects had to ignore all sounds and stay focused on the breath counting task. The auditory oddball protocol was composed of 70-ms pure sounds at 500 Hz for the standard stimuli (70% of the stimuli) and 70-ms pure sounds at 1000 Hz for the oddball stimuli (30% of the stimuli). Auditory stimuli were presented every second at a volume of 72 dB. The oddball stimuli are related to EEG data recordings and analysis and are not analyzed in this report.

Each session lasted 20 min (excluding the time spent filling out questionnaires). Ten sessions of the 20-min breath-counting task were recorded for each subject. Sessions were scheduled every 2–3 days, excluding week-ends, over a period of 5 weeks.

### Ocular data recording

Eye movements and pupil size were monitored with an IView Hi-Speed monocular eye tracker (SensoMotoric Instruments, Berlin, Germany) setup on the subject's dominant eye (right eye for both subjects). This infrared tracking system samples eye position at 240 Hz. Before each session, a 13-point calibration was performed using a 1280 by 1024 pixels calibration picture matching the resolution of the screen. During this calibration step, subjects had to follow a white dot moving randomly across the 13-point calibration sequence. Pupil size was estimated in pixel-square using the area-based algorithm provided in IView X software version 1.7. This algorithm estimates pupil area using the diameter of the disk that best matches the pupil digital image.

### Preprocessing—blink and artifact detection

When considering eyetracking data, blinks result in zero values in the continuous pupil size signal—since the size of the pupil is equal to zero when eyes are closed. Blinks also usually induce non-zero values artifacts at boundaries, as the eyetracker erratically loses track of the pupil when the eyelid partially covers it. This often results in single spikes, spike bursts and sustained spikes periods, which make the exact blink onset and blink offset detection more difficult. Moreover, these types of artifacts are not only present around blinks but may also occur at random places in eyetracking data. The preprocessing had two goals: on the one hand, detect blinks onset and blinks offset as accurately as possible in order to compute blinks duration; on the other hand detect blinks and artifacts in order to interpolate these data portions.

**Figure 2:**
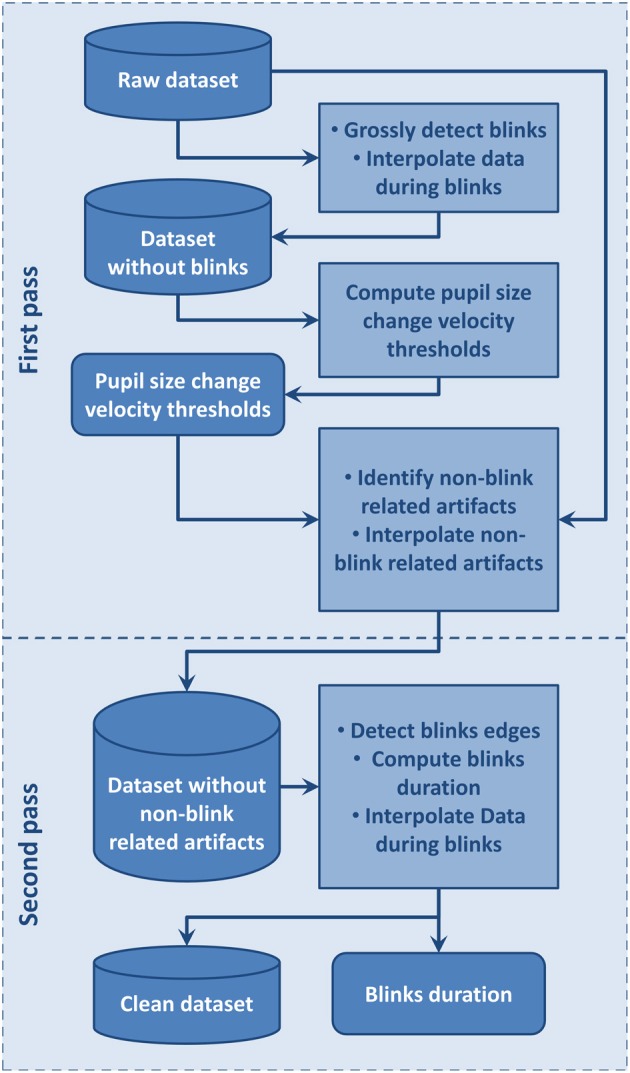
**Flow chart of the automated artifacts and blinks detection procedure used to clean data and compute blinks duration**.

We designed a two-step artifact removal and blink identification procedure inspired by Pedrotti et al. (2011): the first step aims to define statistics of the blink-free signal which is used to detect and remove non-blink related artifact and the second step uses a fine grain blink detection procedure to compute blink duration and interpolate signals during blinks. The automated procedure we developed to achieve this is summarized in Figure 2 and detailed below.

We first distinguished non-blink-related artifacts from blink-related ones (Figure 2). Before applying this procedure, we visually inspected the signal to be sure that no dataset showed periods including artifacts extending over the whole dataset. The blink-free datasets were temporary datasets obtained by removing blink using a conservative procedure where blinks were defined as the period from 100 ms before to 300 ms after a section of pupil-size data equal to 0. This large interval around a blink was used to insure it contained the whole blink period, including the perturbed sections due to eyelid closing or opening, so that pupil size change velocity statistics would not be contaminated by blinks. Blinks separated by a time interval lower than 100 ms were considered as belonging to the same blink and were merged. We then used a pupil size change velocity thresholds specific to each dataset to detect artifacts at blink boundaries such as: lowThreshold = μ − *a*.σ and highThreshold = μ + *a*.σ where μ is the mean of pupil size change velocity over the considered dataset, σ its standard deviation, and *a* a coefficient fixed to 1.5. These thresholds were used on corresponding original datasets where all data points with a pupil size change velocity higher than highThreshold or lower than lowThreshold were flagged as artifact. Artifacts separated by less than 16 ms were merged. Among these artifacts, we then distinguished blink-related artifacts as the artifactual samples situated around zero values. Blink-related artifacts were merged with the blink periods. Non-blink related artifactual values were interpolated in order to avoid interference with the second step where blink onsets and offsets are accurately identified.

For the second step, we used the fine grain blink detection algorithm designed by Engbert and Mergenthaler (2006). This algorithm is tailored for saccade detection but may also be applied to estimate precisely blink onsets and offsets. After detecting blink onsets and offsets, we then interpolated linearly the data during blinks. Blinks separated by less than 100 ms were then merged as a single blink in line with Hupé et al. (2009). This fine grain blink detection procedure allows computation of accurate blink duration and other blink statistics. Once these blink and artifactual periods were defined and interpolated, statistics of pupil size change and gaze position were computed to be analyzed along with the behavioral data.

### Data analysis

#### Epoch selection

After the data were cleaned, we conducted analyses on pupil size, blinks and gaze position. We extracted mind-wandering epochs as the 10-s interval prior to MW button presses. Focusing (F) epochs were defined as the 10-s interval after re-focusing button presses. In order to avoid overlap between MW epochs and F epochs, we discarded all epochs where time interval between successive F and MW button press events was lower than 30 s. We also used subject's answers to the questionnaire regarding the subjective mind-wandering duration to remove epochs where the mind-wandering occurrence was reported to be less than 2 s. Epochs where the questionnaire indicated that the button was pressed by error were not considered in the analysis. In addition, epochs where the subject failed to press the button to indicate that he or she was restarting the breath-counting task were discarded. Data epochs containing more than 40% of interpolated data points (Smallwood et al., 2011) or blinks with duration greater than 4 s within the analysis intervals were also discarded. This resulted in 121 MW and 113 F clean data epochs for S1 and 88 MW and F clean data epochs for S2.

In order to avoid contamination of the pupil data by peripheral motor effects which are known to increase pupil size (Hupé et al., 2009), we excluded data 1 s before and after button press from the analysis. Thus, for the dependent variables linked to blinks, pupil size, and gaze position, the analysis window was defined as a 9 s period ranging from −10 s to −1 s before mind wandering button press for MW epochs and as a 9 s period ranging from 1 s to 10 s after the re-focusing button press for F epochs.

#### Pupil size

For each subject we conducted two types of analysis on pupil size data. For the first analysis, we computed the arithmetic mean and standard deviation of the pupil size for each epoch in each mental state (MW and F). A Wilcoxon rank sum test was then used to assess significant differences between the MW and F states. The second analysis compared the median time courses of pupil size during MW and F epochs. For this analysis, median pupil size was computed at each time point across epochs. A linear regression was then computed on the studied time period in order to extract the estimated slope (a) of the pupil size time course and related Pearson coefficient of correlation (r).

#### Blinks

First, in order to study if blink duration and blink frequency changed with the time spent performing the task, two measures were derived from blinks. Blink duration was computed based on the fine grain blink detection procedure described in section Preprocessing—Blink and Artifact Detection. Blinks with durations longer than 1.5 s were discarded. For each session, mean blink rate and duration were calculated on the continuous data using 1 min sliding windows with 30 s overlap. Mean blink rate and duration were then averaged for all windows across sessions for each subject. Pearson correlation coefficients between mean blink duration and time spent performing the experiment on one hand, and mean blink rate and time spent performing the experiment on the other hand were calculated for each subject.

Second, a contrast analysis between MW and focusing was conducted on the 9-s epochs preceding MW button press and following re-focusing. Mean blink rate and mean blink duration were computed for each type of epoch. As epochs were only 9 s long, blink rate expressed in blinks per minute was approximated by multiplying the number of blinks within each 9 s epoch by 6.67 (i.e., 60 s divided by 9 s). A Wilcoxon rank sum test was performed on each dependent variable to assess differences between conditions.

#### Gaze position

We used the gaze position measure returned by IView X software to assess gaze position. The eye tracker used the orbital pupil position—“Mapped Screen Coordinates” under the IView X software—and returned values in terms of pixel location on the stimulus presentation screen. X and Y coordinates are given in pixels relative to the calibration area used during the calibration procedure. We interpolated the data on these two channels during periods where we interpolated the data on the pupil size channel. Mean gaze position and standard deviation for the X and Y gaze position were calculated for the MW and the F using the same procedure described previously for blink rate and blink duration. The same Wilcoxon rank sum test was used to assess differences between the obtained distributions of values computed on MW and F epochs.

#### Correlation between oculometric data and psychometric measures

We conducted a correlation analysis between oculometric data obtained during the MW episodes and the answers to questionnaires. All non-artifactual data epochs were considered in this analysis.

#### Oculometric data automatic classification

We conducted additional offline analysis to assess if it was possible to classify data based on blink, gaze position and pupil size at the single trial level.

To further control that we were classifying mind wandering data, we considered a third type of data epochs for the classification algorithm: control epochs (CT) defined as a 9 s interval centered in-between MW button press and previous F button press, with no overlap with MW or F epochs. Following the same epoch selection procedure and applying the same constraints than for MW and F epochs, 121 CT clean control epochs could be extracted for S1 and 88 CT clean epochs could be extracted for S2.

We attempted to classify each pair of epoch type (MW vs. CT; MW vs. F; F vs. CT) for each subject using a linear 2-norm soft-margin Support Vector Machine (SVM) using a Gaussian Radial Basis Function kernel with a scaling factor, sigma, of 1. A box constraint of 0.2 was used for the soft margin. The Quadratic Programming method was used to find the separating hyperplan. Data were shifted to zero mean and scaled to unit variance prior to SVM training. MATLAB (The MathWorks) function svmtrain was used to train the SVM.

Evaluation of the classifier accuracy was done using 10-fold cross-validation, where 90% of the data is used for training and 10% of the data is used for testing the classifier. Moreover, for each dataset, we performed the 10-fold cross-validation 100 times—with different partition of the data—to obtain 100 accuracy values, which were used to estimate mean accuracy.

We performed classification on a multivariate dataset composed of 9 variables computed on MW, F, and CT 9 s epochs: “Pupil Size Slope,” “Pupil Size Mean,” “Pupil Size Std,” “Blink Rate,” “Blink Duration Mean,” “Gaze X Position Mean,” “Gaze X Position Std,” “Gaze Y Position Mean,” “Gaze Y Position Std.” We first computed the classification accuracy using the combined variables. We also performed classification on each of these variables independently.

## Results

### Mind wandering behavioral data

Descriptive statistics of mind-wandering episodes for each subjects are shown in Supplementary Table S1. The detail of each session is reported along with the average and standard deviation across sessions. Subject 1 noticed and signaled a total of 264 mind-wandering episodes, whereas subject 2 reported only 160 such episodes. The number of questionnaires indicating unwanted button presses was low for both subjects, with 4 for subject S1, and only 1 for S2. The average MW rate was of one MW occurrence every 45 ± 8 s (mean ± *SD*) for subject 1 and 73 ± 14 s for subject 2.

### Pupil size

**Figure 3:**
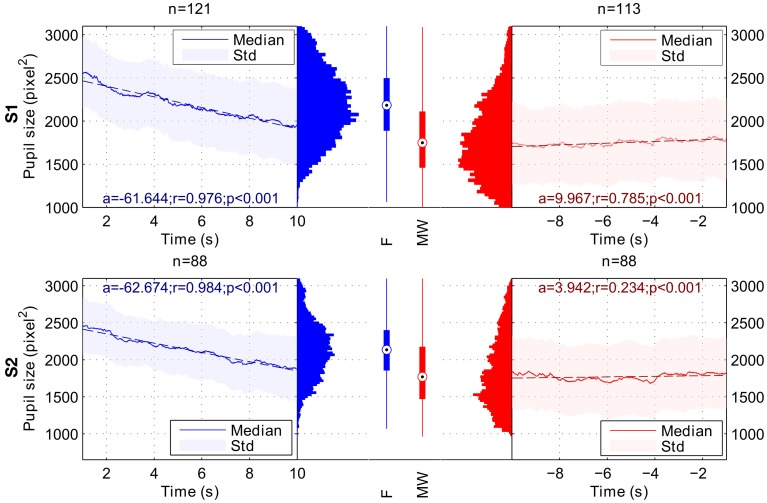
**Variation of pupil size during Focusing (F; in blue) and Mind Wandering periods (MW; in red).** Results for subject 1 are shown on the top row **(S1)**, and results for subject 2 on the bottom row **(S2)**. a, r and *p*-values stand respectively for slope, Pearson's correlation coefficient and *p*-value of the linear regression for the considered time intervals. Linear regressions are represented in dashed lines. In the Focusing period, we observed a significant decrease in terms of pupil size. In the Mind Wandering period, we observed a significant increase in terms of pupil size for both subjects. Global pupil size averaged over time for each interval shows a lower median for MW period.

When comparing the 9-s period following the task refocusing button press with the 9-s period before the mind-wandering button press, Wilcoxon rank tests indicated a significant difference in both pupil size slope and mean pupil size. For both subjects, pupil sizes were significantly smaller during the MW period than during the F period. For both subjects, pupil slope was more negative during the F period than during the MW period. Results are summarized in Table 1. Figure 3 shows the average time course of median pupil size across trials for each subject for the F and the MW periods.

**Table 1:**
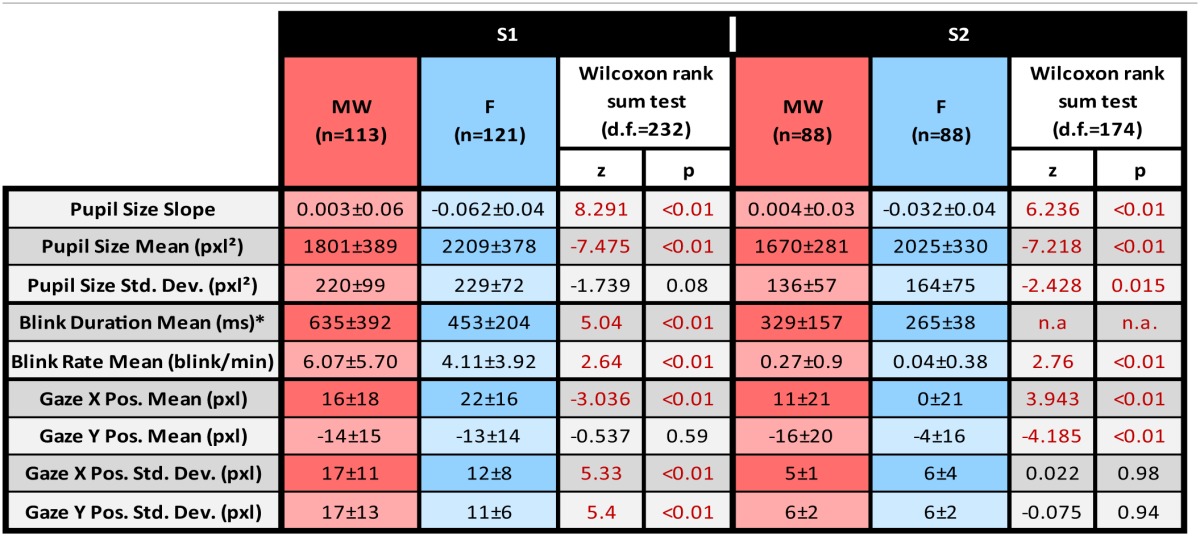
**Mean ± Standard Deviation for each dependent variable**.

Pupil size mean and standard deviation is lower during the mind wandering (MW) compared to the focusing (F) period.

^*^S1: MW(*n* = *162*), F(*n* = *165*), Wilcoxon rank sum test d.f. = *325*; S2: MW(*n* = *11*), F(*n* = *2*).

### Blink frequency

Using Wilcoxon rank sum test, we observed a significant increase in blink rate for the mind wandering period compared to the focusing periods for both S1 (*z* = 2.64, *df* = 232, *p* = 0.008) and S2 (*z* = 2.76, *df* = 174, *p* = 0.006). Blinks were also longer during mind wandering periods compared to focusing periods for both subjects but statistics could only be computed for subject 1 considering the low number of blinks for subject 2 (S1 *z* = 5.04, *df* = 325, *p* < 10^−6^). Statistical results are detailed in Table 1.

Unlike previous reports (Van Orden et al., 2000; Schleicher et al., 2008), we did not observe a clear blink rate increase as subjects spent more time performing the task. A slight increase was found for S1 but it was only marginally significant with a low correlation coefficient (S1, slope = 0.032 blinks per minute, *r*_(2)_ = 0.1, *p* = 0.049). The increase was not significant for S2 (S2, slope = 0.0002 blinks per minute, *r*_(2)_ < 0.001, *p* = 0.98). Blink duration was constant over time for S1 but a weak, albeit significant, correlation between blink duration and time spent on task was found for S2 (slope = 0.00013, *r*_(2)_ = 0.036, *p* = 0.007).

### Gaze position

**Figure 4:**
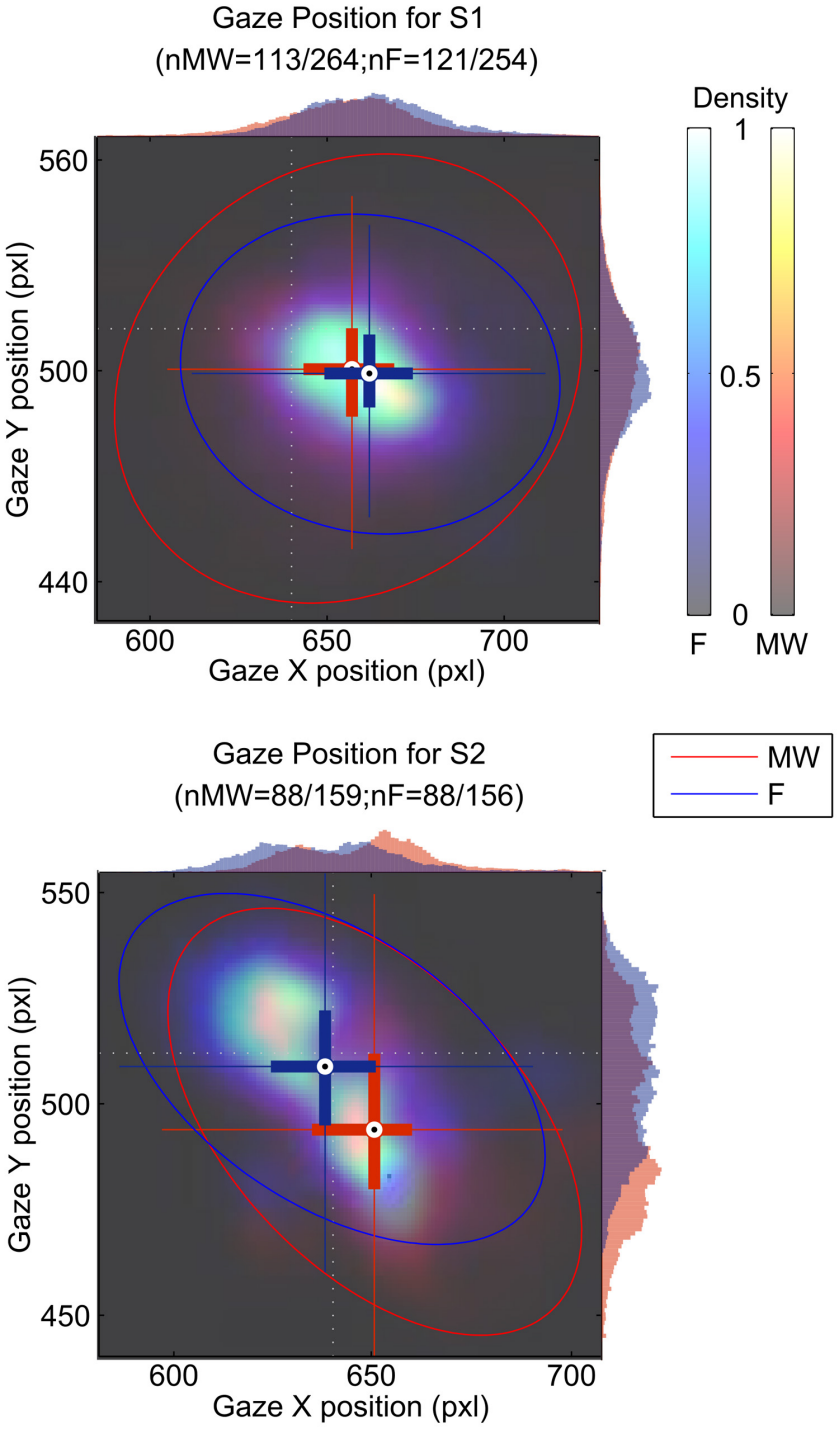
**Gaze position during the focusing period (blue cross and histogram) and the mind wandering period (red cross and histogram).** Gaze position is indicated in pixel (pxl) on both the x and the y axis. The position of the cross indicates the center of gravity for all blink positions for the mind wandering and for the focusing conditions. Ellipses indicate 95% confidence intervals. Histograms of position on the x- and y-axes are also shown on the top and right side of each plot. The light dotted white lines indicate the position of the fixation cross.

We tested if gaze position changed as a function of mind wandering or focusing. Figure 4 represents gaze position density maps computed on mind wandering and focusing epochs. Results are summarized in Table 1.

Differences were found both in terms of horizontal and vertical position of the gaze. X gaze position was significantly different between the MW and F periods for both S1 (*z* = −3.04, *df* = 232, *p* = 0.002) and S2 (*z* = 3.94, *df* = 174, *p* < 10^−4^). Difference in Y gaze position between MW and F epochs was only significant for S2 (*z* = −4.19, *df* = 174, *p* < 10^−4^).

### Classification results

Classification results for the two subjects are summarized in Table 2. Oculometric information used for classification has been ordered according to the averaged accuracy reached when classifying MW vs. F epochs. Classification using the 10 combined oculometric measures considered in Table 2 gave the best results with accuracy over 75% for both subjects when classifying MW vs. F and F vs. CT. Pupil size mean, and Pupil size slope led to the best results for univariate classification of MW vs. F, and CT vs. F. Interestingly, the combined measures provided higher classification accuracy than the best univariate classification for S1, but not for subject S2 for which, the Pupil Size Mean measure alone was better than the combined measures. This could indicate over fitting when using the combined measures. When considering MW vs. CT epochs, performances were close to chance level.

**Table 2:**
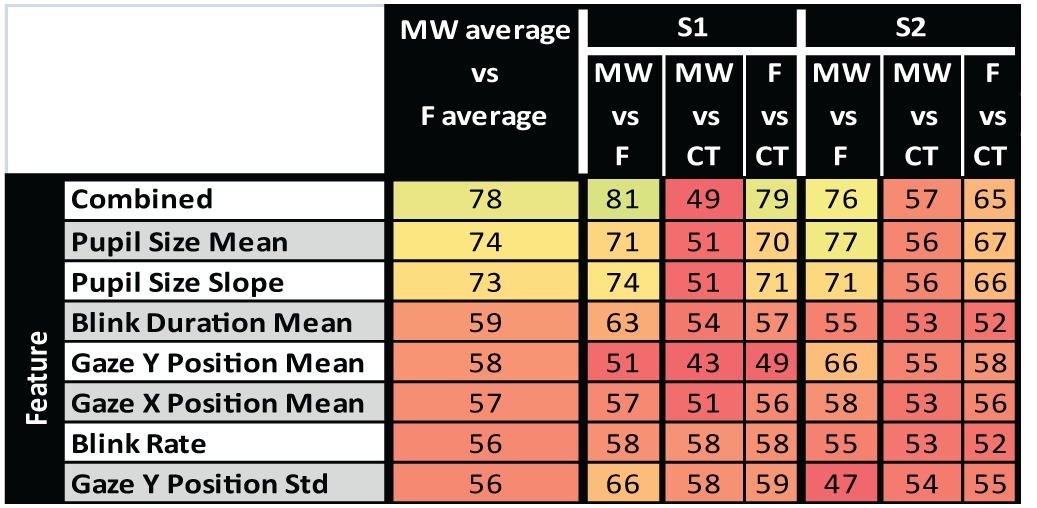
**Results of pairwise classification of MW, CT, and F epochs for single oculometric measure and combined oculometric measures**.

Accuracy is reported as a percentage (50% being chance level). Measures are presented in descending order of accuracy.

### Questionnaire results

Supplementary Figure 3 summarizes the answers of each subject to the questionnaire. We observed that for both subject the majority of mind wandering reports concerned the occurrence of thoughts simultaneous to the execution of the breath-counting task. Most often, there was no time lag between the occurrence of the mind wandering event and its report by the subject. The majority of mind wandering-episodes were reported to last for approximately 10 s.

Regarding thought content of MW episodes, for both subjects the majority of thoughts involved both mental images and inner dialog, and where most often related to events taking place in the surrounding environment. The majority of thought content concerned namely thoughts relative to the task, or the passive observation of a given situation. Thoughts were only rarely related to events taking place far in the past or far in the future.

The emotional content of MW episodes was mostly either slightly negative or neutral. The subject's level of vigilance was mostly either normal (calm) or lower than normal (drowsiness). Subjects differed in the quality of the feelings they reported, with subject 1 experiencing more discomfort than subject 2, while subject 2 seemed more tired than subject 1.

Overall, these preliminary observations provide insights into the characteristics of mind wandering recorded in two subjects during a breath counting task. Collecting data on more subjects would be necessary to draw more general conclusions about the content of mind wandering.

**Figure 5:**
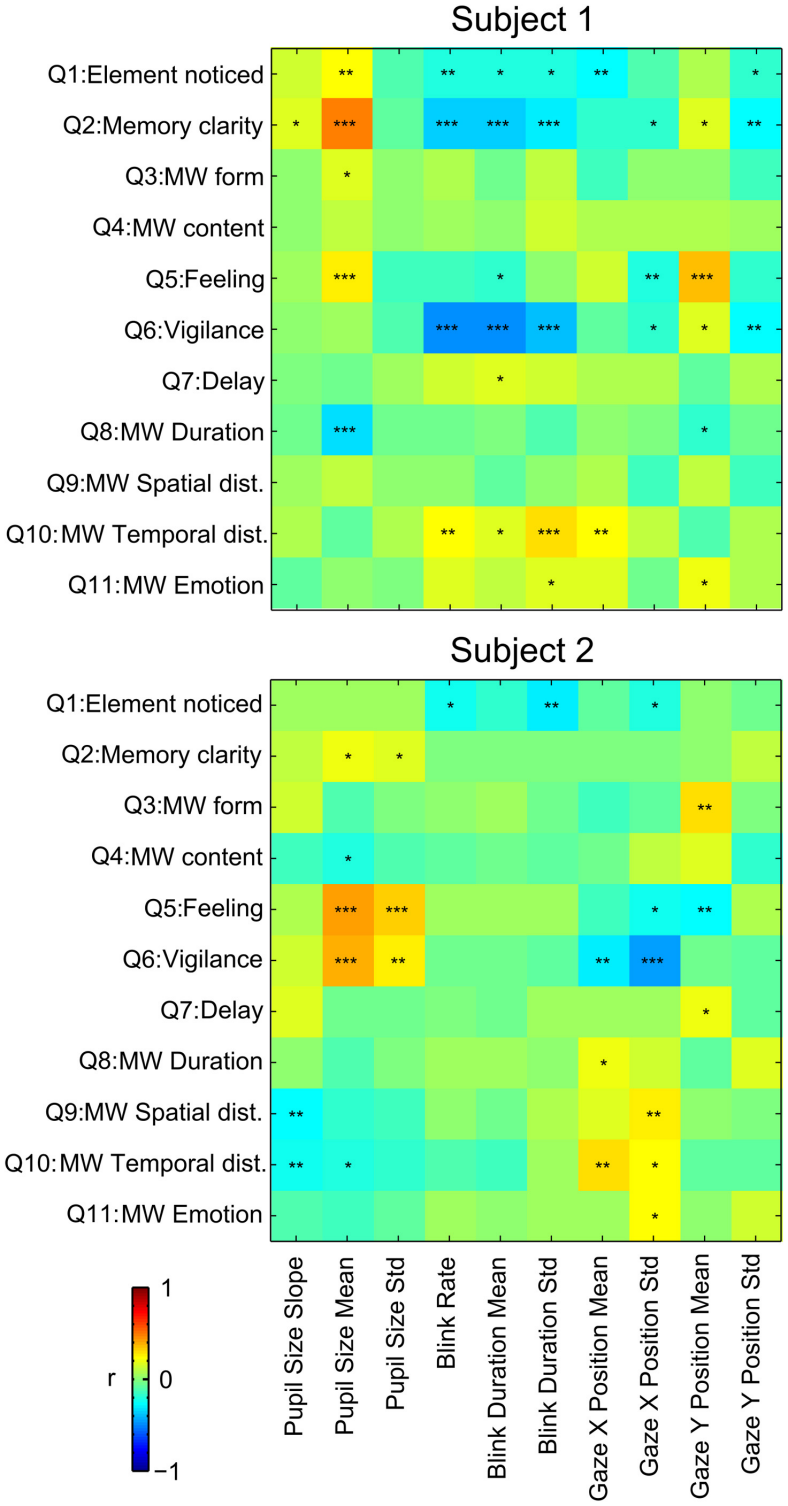
**Correlation between answers to the questionnaires and oculometric measures during the MW epochs.** Pearson's r is reported by color of each cell. ^*^p ≤ 0.05, ^**^p ≤ 0.01, ^***^p ≤ 0.001.

The purpose of collecting this psychometric data was to conduct a correlation analysis between subjects' answers and oculometric measures. Results are summarized in Figure 5.

Memory clarity (Q1) of the mind wandering content and feeling of well being (Q5) were positively correlated with mean pupil size during MW epochs for both subjects. Memory clarity (Q5) and Vigilance (Q6) were inversely correlated with blink rate and duration for subject 1. Absence of results for subject 2 on these measures could be due to the low number of blinks for this subject. X gaze position was positively correlated with the temporal distance (Q10) for both subjects. Most other significant correlations observed were idiosyncratic, and different in the two subjects.

## Discussion

We studied spontaneous mind-wandering episodes in two subjects. To accumulate enough mind wandering episodes, each subject performed 10 sessions of 20 min each over the course of about 5 weeks. Albeit challenging in terms of data collection, this study is a proof of concept that studying self-reported mind wandering episodes along with psychometric data is a valid scientific protocol and may yield interesting results.

### Pupil size

In this pilot study, we have shown that mind wandering correlates with pupil size, gaze position and blink rate. We showed a significant decrease in pupil size during mind wandering. Consistent with our result, Van Orden showed that, in line with reports by Lowenstein and Loewenfeld (1962), Yoss et al. (1970) and McLaren et al. (1992), pupil diameter was typically smaller during periods of increased error.

Previous work has shown that pupil baseline diameter increased during periods where mind wandering was more likely to occur (Smallwood et al., 2011), which is the opposite of what was observed in our data. However, Smallwood's task (2011) involved paying attention to external visual cues and was based on a time-locked paradigm. It is possible that the increase in pupil size in Smallwood's task corresponds to a regulatory mechanism to favor processing of external stimuli during mind wandering. Moreover, their analysis is based on much shorter time windows (a 3–4 s) locked on stimuli. The task in our study does not involve monitoring of visual stimuli. This difference in task could explain the difference in terms of pupil dilation we observed compared to Schooler et al. (2011).

It has also been shown in previous studies that sleepiness results in pupil size decreasing with time (Lowenstein and Loewenfeld, 1962; Yoss et al., 1970). The gradual decrease of the average pupil size can also be related to several factors: time on task and decrease of the cognitive load. Additional data and analyses would be needed to show that the small changes in pupil size induced by mind wandering in our task are independent of these factors.

### Blink rate

We observed an increase in the number of blinks per minute during mind wandering periods in both subjects. A previous study on mind wandering and blink rate during a reading task by Smilek et al. (2010) showed that mind wandering periods contain more blinks than on-task periods. These results and ours are consistent with the hypothesis that blinks might be involved in trade-off modulation between attention to thoughts produced during mind wandering, and attention to task-related stimuli.

### Gaze position

We observed a different average location for the gaze of both S1 and S2 during the mind wandering compared to the focusing period. While the position of the gaze was on the fixation cross for S2 (Figure 4), S1 appeared to be fixating on average on a different point while focusing. It is unclear if this arose because of problems in the software calibration or if S1 actually shifted his gaze during the task. Note that the shift in position only correspond to about 10 pixels, which only represent about 2 mm on the computer screen.

We also observed increased eye movement amplitude as indexed by the standard deviation of gaze position during mind wandering. Consistent with this result, using a reading task, Reichle et al. (2010) observed erratic eye movements immediately preceding self-caught mind wandering episodes. However, they also report longer fixation periods, which could influence the standard deviation of the gaze position during focusing periods. Uzzaman and Joordens (2011) also showed that overall eye movements were slower and less frequent during reports of probe-caught mind wandering. In another study, Smilek et al. (2010) showed that during extended periods of reading, mind wandering episodes contain fewer fixations on the reading material. These results argue in favor of the theoretical claim that the cognitive processes that normally influence eye movements to enhance semantic processing during reading exert less control during mindless reading. Our results are consistent with this hypothesis that control of gaze stability seems to be less efficient during MW.

The type of task to perform may also influence eye movements during mind wandering. When performing a driving task that required visual exploration, He et al. (2011) observed that, while mind wandering, participants tended to focus visual attention more narrowly on the road, which is opposite of what we observed. However, in all studies, the primary task and its oculomotor characteristics, being complex eye movements for reading, wide ocular movements for visual exploration, or on the contrary gaze fixation while counting breath, seem to be executed in a less efficient way, which is consistent with the decreased subjective involvement with the external world during mind wandering.

Note that this last hypothesis is consistent with the decoupling hypothesis where two attentional states are distinguished: the offline and online mental mode resulting from a competition between internal and external information streams for access to a global workspace (Dehaene and Naccache, 2001). During the “online mode,” attention is turned toward external task relevant information, whereas during the “offline mode” attention is turned inward, favoring the inner stream of information and diminishing external information access to the global mental workspace.

### Classification

For both subjects, we observed high classification accuracy of MW, CT, and F epochs using a variety of oculometric measures, pupil size leading to the highest classification performance. However, it is interesting to note that in all cases, classification accuracy of MW vs. F was greater than classification accuracy of F vs. CT, which was itself greater than classification accuracy of MW vs. CT. It thus seems that MW epochs are more similar to CT epochs than to F epochs as classification performances of MW vs. CT are closer to chance level. This might be due to the fact that despite the selection of control epochs in between F and MW events, mind-wandering could have already started in CT epochs. Moreover, definition of MW epochs relies on subjects' capacity to self-detect and self-report their own mind wandering. Therefore, undetected or unreported mind wandering can occur during the considered CT periods. It is worth mentioning that this classification scheme is far from real-time detection of mind wandering occurrence as classification is not done taking random signal epochs or using a sliding time window. However, these results still show that it is possible to use oculometric data to distinguish between focusing or mind wandering data epochs.

### Limits to the self-paced mind wandering protocol

We want to emphasize that, in our protocol, we currently have no way to know if a subject reports less mind wandering episodes because he experiences less mind wandering episodes or because he is less sensitive in detecting mind wandering episodes. The mean mind wandering detection rate across subjects is of about one mind wandering occurrence per minute but shows high variability (mean: 54 ± 38 s). Consistent with our results, Hasenkamp et al. (2012) reported an average of one mind wandering episode signaled every 80 s for the same task duration of 20 min.

In our paradigm, it is also difficult to assess the exact onset of mind wandering periods, which may be variable from one trial to the other. As a consequence, trial averaging is also likely to result in the masking of some temporal structure of mind wandering. Thus, we are mainly studying the offset of mind wandering episodes, as indicated by subjects' button presses.

## Conclusion

While this study is limited in scope since it corresponds to data from only two subjects, it demonstrates that oculometric data can potentially be used as an marker of mind wandering. It also shows that collecting multiple sessions in few subjects may yield reliable and meaningful results for mind wandering research.

## Conflict of interest statement

The authors declare that the research was conducted in the absence of any commercial or financial relationships that could be construed as a potential conflict of interest.

## Supplementary material

Supplementary Figure 1 **Instruction screen displayed at the beginning of each session**.

Supplementary Figure 2 **Questionnaire used to collect phenomenological data.** A sample questionnaire, identical to the questionnaire actually used during the session, was displayed prior to the session start, just after the instruction screen presented in Supplementary Figure 1. Subjects could familiarize with the Graphical User Interface (GUI) and the content of the questionnaire as long as they wanted before starting the experiment session. The questionnaire was filled by pressing buttons on the GUI. The “Cancel” button was used in case the subject clicked on left mouse button by mistake. Button are turned green when clicked. Validate button is displayed only when the subject answered all questions.

Supplementary Figure 3 **Questionnaire answers' statistics summed over the 10 sessions (nS1 = 264, nS2 = 160)**.

Supplementary Table 1 **Descriptive statistics computed on mind-wandering occurrences for each subject, and averaged across subjects. The number of mind-wandering events in this table do not include questionnaires where the subject pressed the cancel button**.
