# Supplementary material for: Oculometric variations during mind wandering
Source: Front Psychol. 2014 Feb 11;5:31. doi: 10.3389/fpsyg.2014.00031 (PMC3920102; doi:10.3389/fpsyg.2014.00031)
Supplement: Supplementary Figure 1 — Instruction screen displayed at the beginning of each session. [file Presentation1.ZIP › 59252_Grandchamp_Presentation_1/Supplementary Table 1.pdf]

Supplementary Table 1: Descriptive statistics computed on mind-wandering occurrences for each subject, and averaged across subjects. The number of mind-wandering events in this table do not include questionnaires where the subject pressed the cancel button.

	session	duration(mn)	nb. MW	mean duration before MW (s)	mean questionnaire duration(s)	total questionnaire duration over session (mn)	total Nb. MW
Subject 1	1	29.65	19	60.30	30.75	9.74	264
	2	31.38	25	46.12	27.65	11.52	
	3	29.11	23	49.14	24.19	9.27	
	4	29.90	26	45.63	23.26	10.12	
	5	33.77	34	33.69	24.71	14.00	
	6	29.38	25	47.39	22.96	9.57	
	7	29.27	21	55.48	26.68	9.37	
	8	28.95	26	44.06	20.93	9.10	
	9	28.77	29	40.95	18.48	8.93	
	10	30.89	36	32.43	18.60	11.19	
	mean	30.01	26.36	45.47	23.59	10.18	
	std	1.49	5.07	8.18	3.78	1.45	
Subject 2	1	29.61	11	103.92	52.65	9.65	160
	2	30.79	14	81.12	46.56	10.86	
	3	28.57	13	82.69	39.93	8.65	
	4	30.69	16	72.45	40.50	10.80	
	5	33.16	20	59.11	39.97	13.32	
	6	30.56	19	61.05	33.80	10.70	
	7	27.81	17	67.36	27.89	7.90	
	8	25.31	14	82.34	23.14	5.40	
	9	25.88	14	77.60	25.31	5.99	
	10	29.78	22	50.70	27.05	9.92	
	mean	29.33	16.09	73.46	35.83	9.44	
	std	2.30	3.30	14.48	9.34	2.31	
Grand total	mean	29.67	21.23	53.71	27.44	9.81	
	std	1.92	6.71	38.21	12.57	1.95	
