# Supplementary figures and images for: Oculometric variations during mind wandering
Source: Front Psychol. 2014 Feb 11;5:31. doi: 10.3389/fpsyg.2014.00031 (PMC3920102; doi:10.3389/fpsyg.2014.00031)

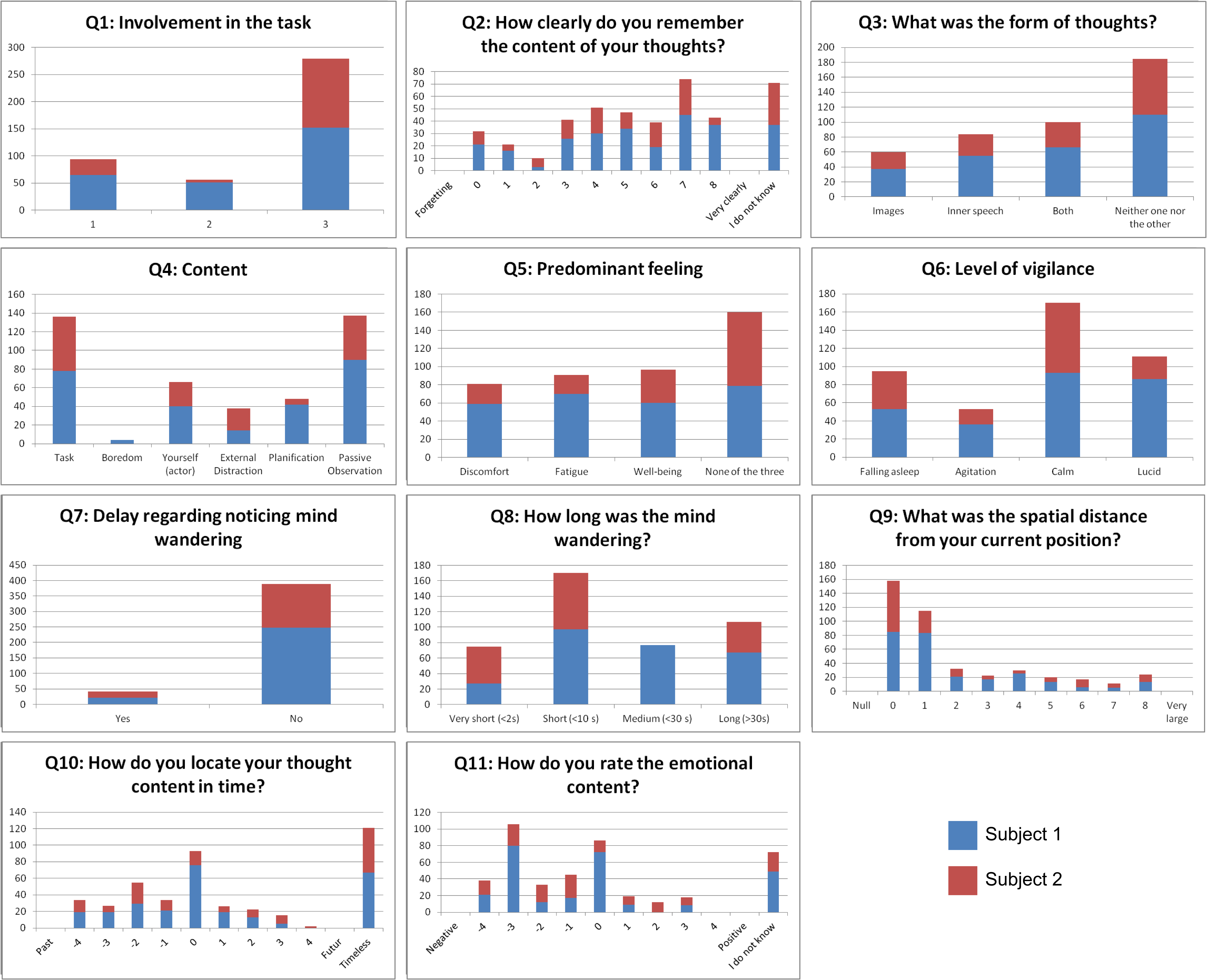

Supplement: Supplementary Figure 1 — Instruction screen displayed at the beginning of each session. [file Presentation1.ZIP › 59252_Grandchamp_Presentation_1/SuplementaryFigure_3.tiff]

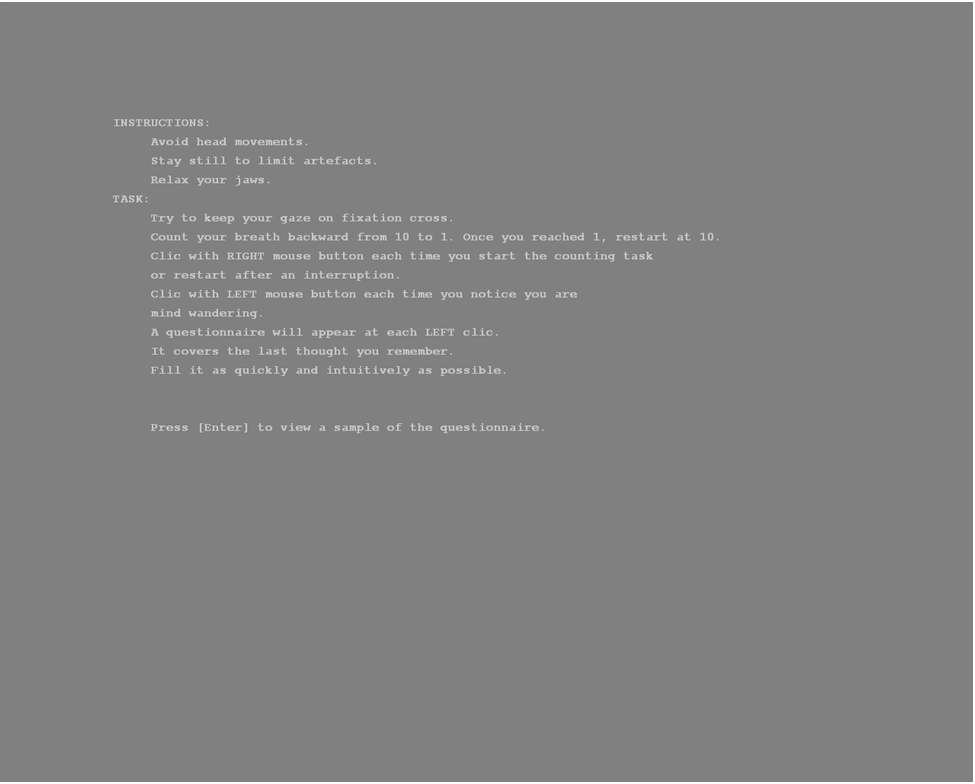

Supplement: Supplementary Figure 1 — Instruction screen displayed at the beginning of each session. [file Presentation1.ZIP › 59252_Grandchamp_Presentation_1/SupplementaryFigure_1.tif]

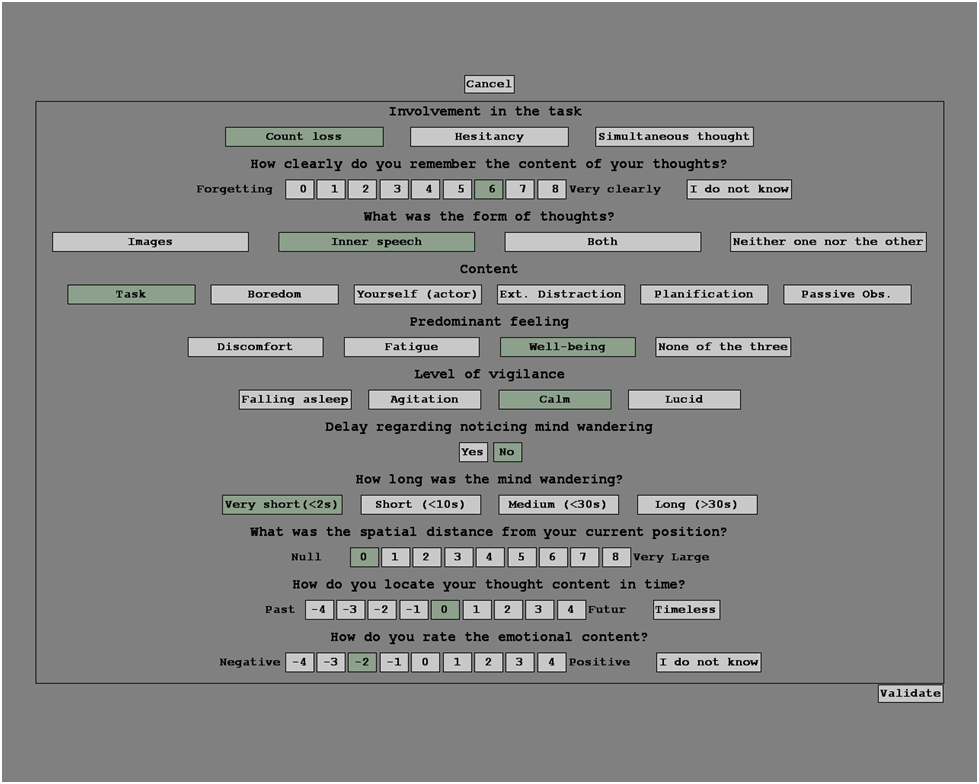

Supplement: Supplementary Figure 1 — Instruction screen displayed at the beginning of each session. [file Presentation1.ZIP › 59252_Grandchamp_Presentation_1/SupplementaryFigure_2.tif]
